# Characterization of Graphene-based FET Fabricated using a Shadow Mask

**DOI:** 10.1038/srep25050

**Published:** 2016-05-12

**Authors:** Dung Hoang Tien, Jun-Young Park, Ki Buem Kim, Naesung Lee, Yongho Seo

**Affiliations:** 1 Faculty of Nanotechnology & Advanced Materials, HMC, and GRI, Sejong University, Seoul 143-747, South Korea

## Abstract

To pattern electrical metal contacts, electron beam lithography or photolithography are commonly utilized, and these processes require polymer resists with solvents. During the patterning process the graphene surface is exposed to chemicals, and the residue on the graphene surface was unable to be completely removed by any method, causing the graphene layer to be contaminated. A lithography free method can overcome these residue problems. In this study, we use a micro-grid as a shadow mask to fabricate a graphene based field-effect-transistor (FET). Electrical measurements of the graphene based FET samples are carried out in air and vacuum. It is found that the Dirac peaks of the graphene devices on SiO_2_ or on hexagonal boron nitride (hBN) shift from a positive gate voltage region to a negative region as air pressure decreases. In particular, the Dirac peaks shift very rapidly when the pressure decreases from ~2 × 10^−3^ Torr to ~5 × 10^−5^ Torr within 5 minutes. These Dirac peak shifts are known as adsorption and desorption of environmental gases, but the shift amounts are considerably different depending on the fabrication process. The high gas sensitivity of the device fabricated by shadow mask is attributed to adsorption on the clean graphene surface.

Graphene^1^,^2^,^3^,^4^ possesses a high carrier mobility and high mechanical strength^5^,^6^,^7^,^8^. These novel properties make it a promising new material for replacing Si in the semiconductor industry. It has been previously shown that graphene is strongly influenced by doping from contaminated interfaces. Therefore, practical applications of graphene based field-effect-transistors (FETs) require reliable and suitable supporting substrates. The effects of various substrates on the electrical properties of graphene devices have been widely studied^9^,^10^,^11^,^12^,^13^,^14^, especially doping effects of the substrate^14^,^15^,^16^,^17^. The quality of graphene on an Si/SiO_2_ wafer is limited by scattering from impurities and charged surface states, roughness, and optical phonons of the SiO_2_ surface. Also, an inhomogeneity of electron-rich and hole-rich puddles arises due to the charged impurity and substrate-induced disorder. This inhomogeneity^3^,^18^ leads to local variations of the electrostatic potential at the SiO_2_ surface, and trapped charges^19^,^20^ result in an uncontrolled local doping effect on the graphene sheet. Recently, hexagonal boron nitride (hBN) has been adopted as an ideal substrate for graphene-based devices due to its smooth surface and crystal similarity to graphene^14^,^17^. However, the details concerning the effect of the substrate on the electrical properties of graphene are still debated. In our work, the electrical properties of the graphene on SiO_2_ and hBN were investigated under various conditions.

Employing e-beam or photolithography while patterning electrical contacts unnecessarily exposes the graphene surface to chemicals (photoresist, e-beam resist, or solvents). These chemical residues have been reported to be nearly impossible to completely remove, thereby degrading graphene device quality^21^. In order to create a perfectly clean graphene surface, the lithography free method was adapted without using polymers and solvent in this study. We used a transmission electron microscopy (TEM) grid as a shadow mask to fabricate electrical contacts avoiding the exposure of the graphene to lithography resist and solvent chemicals. Though other materials such as an ultrathin quartz filament, hard Si shadow mask, and micro soldering have also been reported^22^,^23^,^24^, the TEM grid as a shadow mask has an important advantage because it is commercialized and thus easy to obtain. The alignment process for the TEM grid reported in this paper is accurate at the sub-micron scale and can be used for general purposes. As the device fabricated with a TEM grid is clean without the polymer residue, the influence of substrates on a graphene device was investigated excluding doping effects from the residue. Also, the gas adsorption and desorption study was motivated by the fact that the clean graphene surface should be very sensitive to a gas environment.

## Experimental Details

The graphene channel of FETs was supported on Si/SiO_2_ substrates, where p^+^ doped Si (*ρ* ∼ 3 × 10^−3^ Ωcm) was used as the bottom gate electrode and SiO_2_ (300 nm thermal oxide) as the gate dielectric. First, the Si/SiO_2_ substrates were sonicated in acetone, then in isopropanol, and later rinsed by DI water. Highly oriented pyrolytic graphite (Grade 300, Graphene supermarket) and natural Kish graphite were used to produce graphene flakes by mechanical exfoliation via adhesive tape (Scotch Tape, 3 M, Inc). The graphene based FETs were also fabricated on the hBN substrate. For graphene on hBN fabrication, hBN flakes were first deposited on the Si/SiO_2_ wafer using the mechanical exfoliation method. Then, a microscope was utilized to select one appropriate hBN flake, which was the substrate for fabricating graphene FET. A graphene layer was transferred to the hBN substrate using the dry transferring method^14^,^25^. All hBN and graphene flakes were produced using the Scotch tape method. After the graphene flake was transferred to the hBN substrate, electrical contacts were deposited using TEM grids as shadow masks in the evaporation process.

Electron-beam evaporation was used to make electrodes with Ti (1 nm) followed by Au (80 nm) for electrical contacts at both ends of the graphene FET channel (‘source’ and ‘drain’). The electrodes were evaporated onto the graphene through square holes in a commercial grid for transmission electron microscopy (TEM), which was used as a shadow mask. Two kinds of TEM grids (SPI Supplies Structure Probe, Inc.) were employed, and the specifications are listed in Table 1 of the Supplementary Information. For the first evaporation, a fine grid of 5 μm bar width was used, and then another large grid of 25 μm bar width was employed to extend the electrical contact pad size, which was connected to wires. In order to create a long contact pad, some bars of the large TEM grid were cut with a razor blade. (Supplementary Figure S2).

**Figure 1:**
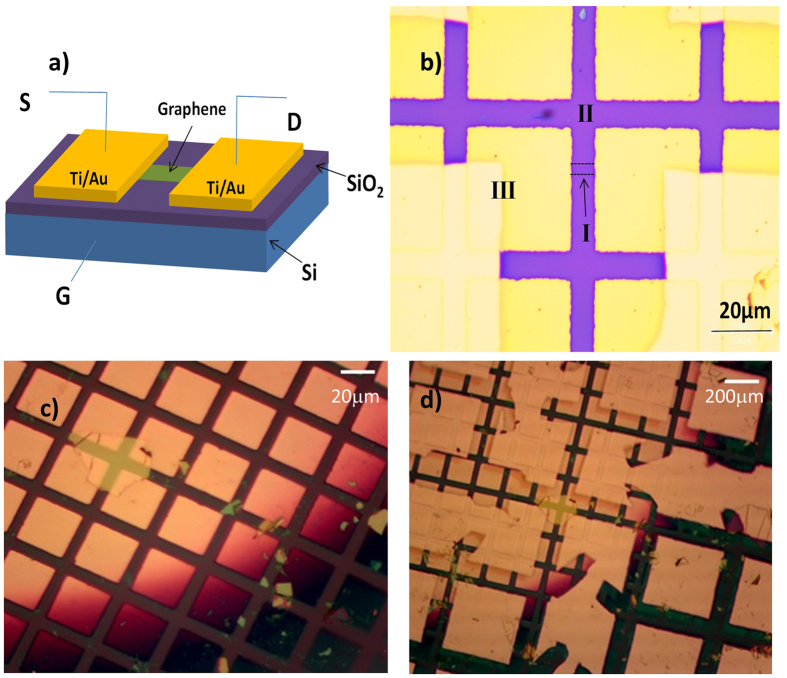
(**a**) Graphene device supported on SiO_2_ with underlying doped Si serving as the back gate, G; S and D refer to the source and drain contacts, respectively. (**b**) Optical micrograph of a graphene device fabricated with TEM grids as a shadow mask. A single graphene layer (I) (∼3 μm wide indicated with dashed lines), SiO_2_ dielectric (II), and Ti/Au electrodes (III) are shown. (**c**) The graphene on hBN device after first evaporation using fine TEM grid as a mask. (**d**) The device after second evaporation using a large TEM grid as a mask, where larger contact pads were used as wiring to execute the measurements.

A schematic drawing of our back-gated graphene FET supported on a SiO_2_/Si substrate is shown in Fig. 1(a), together with an optical micrograph of the device (Fig. 1(b)). The optical image shows the square array of Ti−Au contacts deposited covering two ends of a rectangular graphene flake of dimensions ∼7 × 3 μm^2^. To pattern the electrodes, we used a polydimethylsiloxane (PDMS) slab as a supporting layer carrying the TEM grids. The PDMS slab has a dimension of 5 × 5 × 1 mm^3^ with a punched hole (1–2 mm dia.), where the center of the TEM grid was located. After that, the PDMS slab was affixed to a glass slide. The slide with the PDMS slab was inverted and attached to a micromanipulator. The micro manipulator was then utilized to position the TEM grid covering the chosen part of the graphene flake on the hBN substrate. Using a micromanipulator while monitoring the area through an optical microscope, the TEM grid was carefully positioned on the graphene flake of interest (i.e., natural flakes of approximately rectangular shape) so that one of the TEM bars covered the graphene, leaving its opposite side ends uncovered between the bar of the mask. After the first evaporation, a square array pattern of the fine TEM grid was created as shown in Fig. 1(c). To elongate the electrodes, the large TEM grid was used for the second evaporation. The method to place the large grid over the chosen location was the same as the method used for the fine TEM grid. The alignment was performed accurately so that the bars of the second grid covered the graphene channel area. Figure 1(d) displays the final image of the graphene based FET supported on the hBN substrate.

## Results and Discussions

As our device fabrication process involves shadow-masking to make electrical contacts, this method has the crucial advantage of being a resist-free process. After finishing the fabricating process, no other treatments (e.g. heat treatments^26^ or current annealing^27^) were employed for our graphene FET devices. The fabricated graphene devices were exposed to ambient conditions for several days before electrical characterization. Under ambient conditions the electrical measurements always showed that the samples were p-type doped. That is, the Dirac peaks in *R*_*tot*_ versus *V*_*g*_ curves were found at a significantly positive *V*_g_ (*i.e.*, *V*_g_ > +50 V) for most of the fabricated samples. This positive Dirac peak shift of the graphene devices on the SiO_2_ substrate has been observed in other studies^1^,^28^,^29^. On the other hand, this p-doping state was reversed into n-doping under vacuum conditions with thermal annealing by others^30^. This reversible process implies that the p-doping on graphene originates from the adsorption of ambient gas molecules removing electrons from the graphene.

**Figure 2:**
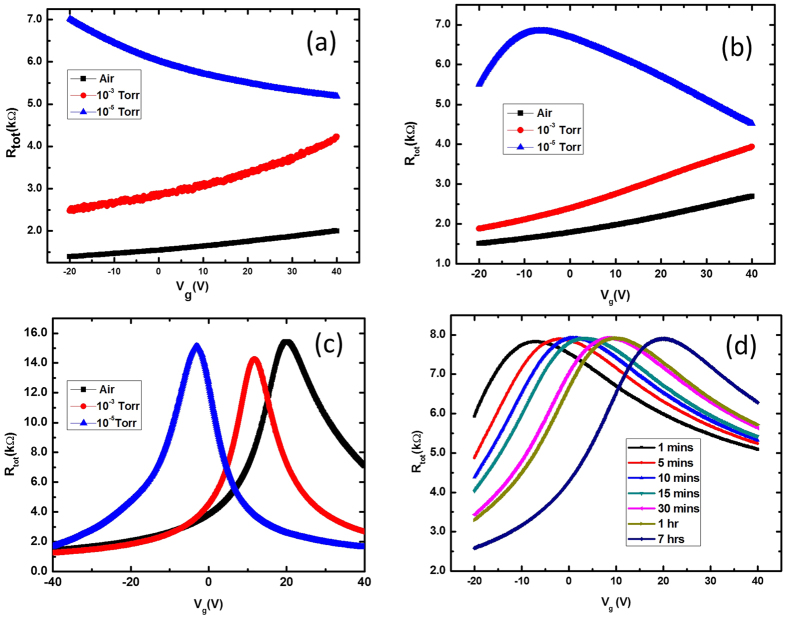
R_tot_ vs V_g_ of the graphene devices on (**a**) SiO_2_ and (**b**) hBN made by using a shadow mask at room temperature. Dirac peaks were shifted more than 60 V depending on the air pressure. (**c**) R_tot_ vs V_g_ of the graphene on SiO_2_ device at room temperature of which electrical contacts of this FET were fabricated using e-beam lithography. (**d**) *R*_*tot*_ vs *V*_*g*_ were measured at different times after venting for the same FET as was used for the data (**a**).

The graphene channel (10 μm long and 3 μm wide) supported on the SiO_2_ device was measured under ambient conditions at room temperature as seen in Fig. 2(a). The Dirac peak was located at positive *V*_*g*_ ≫ +40 V under ambient conditions. When the chamber pressure was decreased to 1 × 10^−3^ Torr at room temperature, the peak shifted to smaller *V*_*g*_, but the value of the *V*_*g*_ at the Dirac point was still over +40 V. (For estimation of Dirac peak shifts, see supplementary information, Fig. S8) The sample was then kept at 1 × 10^−3^ Torr for 7 hours at room temperature and measured again, and the Dirac peak was still greater than +40 V. While the graphene device was heavily p-doped under ambient conditions and at low vacuum (1 × 10^−3^ Torr), the doping polarity was completely reversed at a pressure of 1 × 10^−5^ Torr without heating. The Dirac point was shifted down to *V*_g_ < −40 V at high vacuum conditions. This n-doping source in vacuum should be different from p-type dopant at ambient. The p-type dopants acting as electron acceptors are gases (or water vapor) adsorbed on graphene surface and at the interface between the graphene substrate under ambient conditions. The adsorbates were desorbed in a high vacuum without annealing, and the p-doping effect disappeared.

The Dirac peak at a positive gate voltage implies that the major carrier holes dominate the electrical transport at zero gate voltage. It is known that the defects at the SiO_2_ surface act as gas adsorption sites to adsorb gas and water molecules^31^. Graphene can be doped by an electric field or chemical compounds^32^. The adsorption of doping species, as well as the electric field, can transform graphene with a zero-band-gap completely into electron, or completely hole, conductors. It is known that H_2_O, O_2_, and NO_2_ lead to the p-type doping of graphene, while ammonia leads to the n-type^10^,^30^,^32^. The Dirac point of the graphene FETs was shifted by the p-doping effect of the water in air^33^ and returned to its original state when water vapor was removed, which was reported previously^34^. Judging from the fact that the resistance at 10^−3^ Torr in Fig. 2(a) is higher than that in air, the water molecule is suspected to be the main dopant of p-doping in air. The water molecule acts as an acceptor, and it can receive an electron from graphene by the reaction


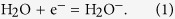


Usually, under ambient conditions oxygen and water molecules can be adsorbed on the SiO_2_ surface or directly adsorbed on the graphene surface^35^, and the physical adsorption disturbs the balance of concentration between electron and hole. Oxygen (O_2_) and NO_2_ gases could serve as additional dopants existing in air, as they also adsorb weakly to graphene, acting as an electron acceptor^33^,^36^.

To study the effect of the underlying substrate on doping graphene, hBN was employed as a substrate along with SiO_2_. The graphene FETs supported on hBN of a few nm thickness were fabricated by the same method with TEM grids as shadow masks. The p-type doping behavior also occurred in the graphene on hBN as shown in Fig. 2(b). In ambient conditions the Dirac peak of the device occurred at positive *V*_*g*_ > +40 V at room temperature. When the chamber pressure decreased to 1 × 10^−3^ Torr, the peak shifted to lesser V_g_, but the *V*_*D*_ was still over +40 V. The results were very similar to those obtained with graphene on SiO_2_. The sample was then kept at 1 × 10^−3^ Torr at room temperature for 7 hours and measured again. The Dirac peak was still higher than +40 V, confirming that the desorption process does not depend on the duration time but the pressure mainly. This p-doping effect of the graphene on hBN can be explained by adsorbed gases and water moisture from the surrounding environment, which is similar to the graphene on SiO_2_. The mobility of this sample was estimated as 1.96 × 10^3 ^cm^2^/V/s from the Drude formula.

For comparison, the other graphene devices (6 μm long and 3 μm wide) were fabricated depositing the metal contact pads using e-beam lithography. The *R*_*tot*_*/V*_*g*_ curves of the sample measured under ambient conditions at 1 × 10^−3^ and 1 × 10^−5^ Torr at room temperature are depicted in Fig. 2c. The Dirac peak at ~25 V in ambient conditions shifted to ~10 V at low vacuum, and ∼−5 V at high vacuum. The mobility of this sample was estimated to be 1.69 × 10 cm^2^/V/s, which is somewhat lower than that of the device fabricated by the TEM grid. This device also displayed a p- doped state in air, but the p-doping effect was weaker than that of the devices fabricated using the shadow mask. This difference may be due to the residue remaining on the graphene surface (See Supplementary Fig. S4) and the interface between graphene and substrate^37^. The polymer and solvent residue attached to the surfaces can act as obstacles preventing adsorptions. Therefore, the graphene layer is less p-doped than that of the devices fabricated without resist. The p-doping behavior of graphene based FETs was recovered after venting and exposure to ambient conditions.

The *R*_*tot*_ versus back-gate bias *V*_*g*_ of the graphene on SiO_2_ device used in Fig. 2a at different times after venting at room temperature is shown in Fig. 2d. As can be seen in this figure, the Dirac peak shifted toward the positive back-gate voltage region. As time passed, the p-doping effect became stronger since more gas and water molecules either adsorbed onto the graphene surface or permeated into the graphene/substrate interface. Judging from the slowness of the process, taking more than several hours, the permeating into interface rather than surface adsorption of water molecules was more probable. The time constant from exponential decay function fitting was estimated to be 0.76 hr, and a similar behavior has been reported by others^34^.

**Figure 3:**
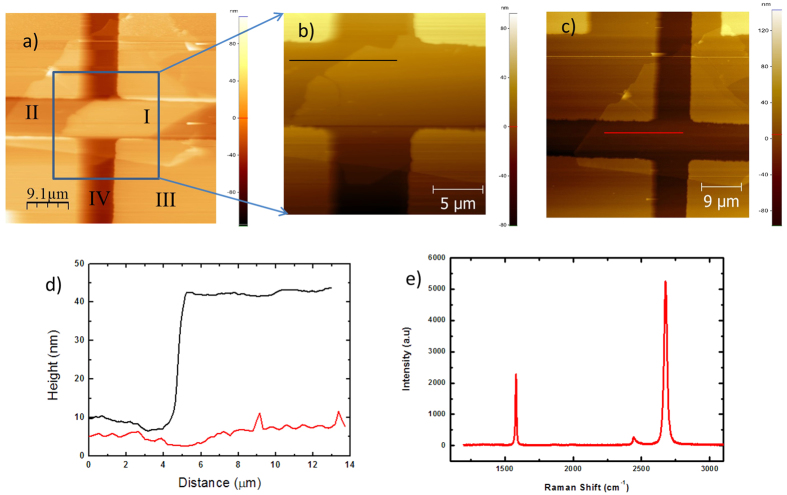
(**a**) AFM topographic image of the graphene on hBN sample in ambient conditions with scan size 45 μm. (**b**) The graphene area on top of hBN was rescanned in air. The shape of graphene was clear with increased thickness as absorbing gases or moisture from surrounding. (**c**) AFM image of the graphene on hBN measured immediately after exposing air from in-vacuum at 1 × 10^−5^ Torr with scan size 40 μm. In this image, single-layer graphene was invisible as the condensed moisture was removed in vacuum. (**d**) The height profiles along lines in (**b**,**c**) are shown, respectively. The thickness of the graphene layer including condensed moisture was ~30 nm. (**e**) Raman spectroscopy data were measured to confirm it as a single-layer graphene.

The sample was left exposed to ambient conditions, and then AFM images of the sample were taken. Figure 3a shows the AFM images of the graphene on hBN fabricated using shadow masks. In these pictures the graphene layer transferred onto hBN substrate is noticeable. The following areas can be identified in the AFM image: (I) single graphene layer, (II) hBN substrate, (III) Ti/Au electrodes, and (IV) SiO_2_ dielectric. This graphene layer was confirmed as a single layer graphene (SLG) by Raman spectroscope, so the thickness should be about 0.3 nm. However, the thickness of the graphene layer was estimated to be ~30 nm (Fig. 3d). Thus, the graphene flake protruded from hBN substrate, presumably attracting gases or moisture from surrounding air. We insist that the water vapor was not adsorbed only on the surface of the graphene layer but condensed at the interface between the graphene and hBN substrate^38^,^39^. These condensed molecules caused a p-doping effect on electrical characteristics of the graphene FET.

To confirm the degassing of the adsorbed gases and water vapor due to vacuum pumping, AFM scanning of the same sample was performed after it was placed in vacuum at 1 × 10^−5^ Torr for 8 hours. The graphene on hBN substrate became almost invisible in the AFM images (Fig. 3c). There is a huge difference between Fig. 3a,c. The thickness of the graphene estimated from the line profile (Fig. 3d) was about 1 nm, and it was confirmed to be a single-layer graphene from Raman spectroscopy data (Fig. 3e). This is too small compared with 81 nm (i.e., the thickness of the Ti/Au electrodes). Hence, the graphene on the hBN substrate is almost invisible in the AFM images. At 1 × 10^−5^ Torr and room temperature, the graphene base FETs became heavily n-doped and the Dirac peak was shifted toward the negative back-gate voltage region. The N-doping effect occurred when graphene adsorbed gases or chemical elements which acted as electron donors, but in high vacuum (1 × 10^−5^ Torr) adsorbed gases were removed from graphene. A high vacuum environment, along with heating, has been used to degas water vapor and adsorbed gasses on both the graphene surface and the interface between graphene and substrate^33^,^40^. Therefore, the n-type graphene doping at high vacuum was not due to adsorbing gases.

Unexpected n-doping behavior of graphene on hBN was occasionally found even on high quality samples by others, but no clear theoretical explanation has been suggested beyond attributing it to contamination^14^,^41^. The n-doping effect of graphene at a high vacuum level may be related to a charge transfer from substrates. It was found that epitaxy graphene on silicon carbide (SiC) can be n-doped by the substrate, that is, the Dirac point of graphene shifts below the Fermi level^42^. In contrast, gold with a higher electron affinity, shifts the Dirac point into the unoccupied states inducing p-type doping of epitaxy graphene^42^. Romero *et al.* suggested that the observed n-type behavior is associated with a low work function of SiO_2_ substrate (W = 3.03 to 3.41 eV)^43^ relative to graphene (W_g_ = 4.23 to 4.48 eV)^17^. The intrinsic exchange of charge between SiO_2_ substrate and the graphene layer depends on atomic configurations of SiO_2_^43^. In their model, the SiO_2_ has an amorphous structure and transfers electrons to graphene. Additionally, the number of electrons transferred from the SiO_2_ substrate to graphene increases rapidly as the graphene is close to SiO_2_. In our work, the n-doping effect of graphene at high vacuum occurs with not only graphene on SiO_2_, but also graphene on hBN substrate. Few-nanometer-thick hBN was not enough to shield the low surface potential of SiO_2_, resulting in the n-doped graphene, because the Fermi level shift in graphene, due to hBN with several layers, was approximately 0.15 V from density functional theory^17^. On the other hand, Caillier *et al.* reported an identification of a strong contamination source for graphene in vacuum systems^44^, where strong n-doping of graphene was found due to chemical species generated by common ion high-vacuum gauges. As our samples were clean and degassed, the n-doping effect on graphene was conspicuous due to chemicals generated by the vacuum ion gauge, as well as the low work function of the substrate.

**Figure 4:**
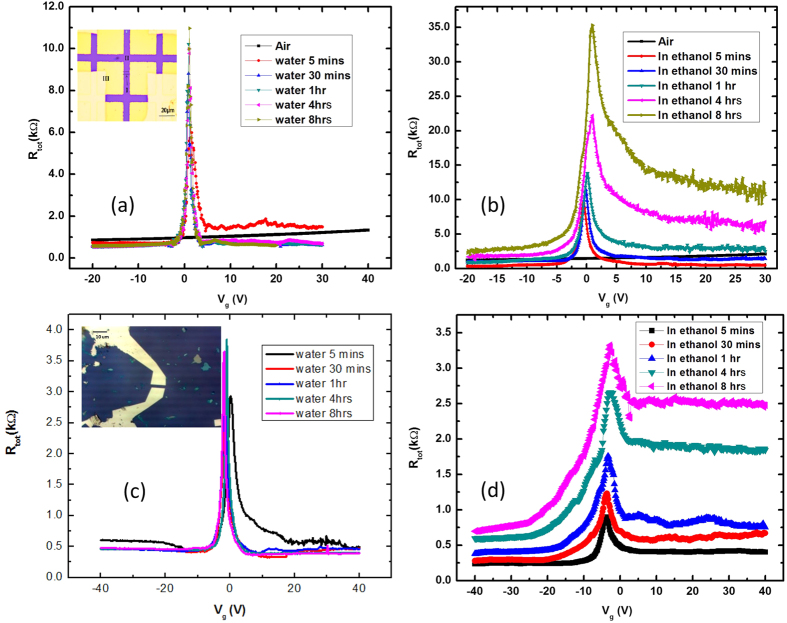
Relationship between *R*_*tot*_ and *V*_*g*_ of the graphene on SiO_2_ device fabricated using TEM grids was measured (**a**) in water and (**b**) in ethanol, as well as in air. Also, *R*_*tot*_ and *V*_*g*_ of the graphene on SiO_2_ device fabricated using e-beam lithography was measured (**c**) in water and (**d**) in ethanol. All measurements were performed at room temperature.

We also conducted a Dirac point measurement of graphene FETs in different liquid environments in order to confirm the effects of the adsorbed water layer. The relationship between resistance *R*_*tot*_ and *V*_*g*_ of the graphene on SiO_2_ measured at room temperature in water is shown in Fig. 4a. While the sample was dipped in water for 8 hours, measurements were conducted at different times. Electrical contacts of this FET device were fabricated using the TEM grid method. In ambient conditions graphene was heavily p-doped as prepared. After dipping the sample into water for 5 minutes, the Dirac peak of the sample shifted to zero back-gate voltage. The Dirac peak changed very little, even while submerged in DI water for 8 hours.

The same measurement was performed in ethanol as shown in Fig. 4(b). After dipping in ethanol, the Dirac peak immediately shifted near a zero back-gate voltage, but the resistance was slowly increased as time passed. The slow increment of resistance can be explained by the contamination from the adhesive polymer remaining in the exfoliation process, as ethanol can dissolve the adhesive. The contaminants in the surrounding area were moved onto a graphene surface, and the resistance of the graphene was increased due to more scattering centers. We observed the same behavior in the graphene on SiO_2_ with electric contact pads fabricated using e-beam lithography as shown in Fig. 4(c,d). Moreover, graphene on the hBN device also displayed similar behavior. (See Fig. S8).

These electrical characteristics of the graphene based FETs in liquid dielectric environments can be explained by the change of effective back-gate capacitance^45^. Ponomarenko *et al.* found that the top dielectric can induce significant changes in the back-gate capacitance due to the finite width of graphene devices^46^. Xia *et al.* confirmed that the back gate capacitance was increased by up to 2 orders of magnitude in water, by measuring the Hall-effect, which was dependent on the dielectric constant of the medium^45^. Thus, the narrowing of the Dirac peaks in our devices can be attributed to a large increment of gate capacitance rather than mobility enhancement. Due to this large change in gate capacitance, the Dirac peak shift by water adsorption was not able to be confirmed in a liquid environment.

## Conclusions

A lithography free method was demonstrated to eliminate polymer and solvent residues remaining on the graphene surface. In this study, we used a shadow mask to fabricate graphene based FET. The commercial TEM grids were utilized as a shadow mask for evaporation to deposit electrical metal contacts. Patterning with a shadow mask eliminates the use of polymers and solvents that are unavoidable in e-beam lithography or photo lithography. Our clean graphene device fabricated by shadow mask patterning indicated high sensitivity in both gas and liquid environments.

The electrical properties of the graphene based FETs on SiO_2_ and on hBN were investigated in various levels of vacuum, in ambient conditions, and in liquid. We found that graphene FETs were heavily p-doped as prepared in air, and this p-doping in electrical behavior of graphene was reversed into n-doped during vacuum pumping at room temperature. During the vacuum pumping process, the absorbed gases on the graphene surface under ambient conditions were able to be removed without annealing. From the AFM data showing inflated graphene and slow adsorption and desorption processes, the water is supposed to be a main source of the doping effect. The reversible changes at room temperature in the adsorption and desorption cycling imply the occurrence of physisorption rather than chemisorption. As water possesses a much lower saturation vapor pressure at room temperature, the majority of adsorbates should be water molecules. The n-type doping effect was found at 1 × 10^−5^ Torr in clean graphene surfaces supported on both SiO_2_ and hBN at room temperature. This n-doping effect from the substrates was explained by the work function of SiO_2_ being lower than graphene. The liquid environment measurement showed narrowing of the Dirac peak, which was attributed to the increment of the gate capacitance. Most results measured in our sample were similar to those reported previously by other groups, but our results were more sensitive to the environment as the sample was prepared in dry conditions without using polymer resist. As a result, the dominant doping source for the graphene device was not polymer residue, but water in air. The clean surface of graphene adsorbs surrounding water molecules strongly, while conventionally processed devices should be covered with polymer residue on top of graphene, preventing the adsorptions. Thus, the device fabricated by the resist-free method showed higher sensitivity in environmental gas than the device with polymer resist. By encapsulating the graphene in vacuum after the metal deposition using a shadow mask method, a high quality graphene device can be fabricated.

## Methods

### Sample preparation

Natural Kish graphite (Grade 300) was mechanically exfoliated on a Si wafer. The TEM grid was attached at the center of a hole punched in a PDMS slab. The TEM grid was carefully positioned using a micro-translator in the xy-plane, to locate the TEM grid into the target position. After alignment, z-directional contact made the PDMS slab stick to the wafer, due to its adhesive property. The adhesion was sustained in a high vacuum evaporation process, where Au contact pads were deposited through square holes of the TEM grid.

### Measurements

The transport characteristics of the devices with three terminals were measured in air and vacuum at room temperature. During the vacuum pumping process, the absorbed gases on the graphene surface were able to be removed under ambient conditions in the laboratory. The semiconductor characterization system (Keithley, 4200-SCS) was employed for accurate electrical measurements. A bias voltage was applied between the source and drain terminals of the graphene channel. The Si substrate was used as a global back-gate; this bias field controls the carrier concentration and polarity in the graphene layer. The voltage applied to gate terminal was swept continuously during the measurements. The output signal was converted to the resistance of the device and then used to plot the graph. The Dirac point of the device was determined from the graph. AFM measurements were performed using a commercial AFM (XE-100, PSIA Co.). Pt/Cr (Multi75E-G) coated cantilevers with stiffness 3 N/m were utilized in noncontact mode for the AFM imaging.

## Additional Information

**How to cite this article**: Tien, D. H. *et al.* Characterization of Graphene-based FET Fabricated using a Shadow Mask. *Sci. Rep.* **6**, 25050; doi: 10.1038/srep25050 (2016).

## Supplementary Material

Supplementary Information
