# Supplementary material for: Characterization of Graphene-based FET Fabricated using a Shadow Mask
Source: Sci Rep. 2016 May 12;6:25050. doi: 10.1038/srep25050 (PMC4864380; doi:10.1038/srep25050)
Supplement: Supplementary Information [file srep25050-s1.pdf]

Supplementary Information

1. Details of fabricating graphene based devices using TEM grids

a. Making PDMS

In our study, PDMS (Polydimethylsiloxane) slab was utilized as the supporting substrate for carrying and aligning the TEM grid. The PDMS slab must be flat with uniform thickness as much as possible in order to let the TEM grid close to the graphene and wafer for precise alignment. For this purpose we have made molds by using microscope glass slides (Fisherfinest™ Premium Plain Glass) (Fig. S1a) with 1 millimeter thickness. The mold consists of two or several glass slides which are separated by two pieces of the same glass slides on both edges (Fig. S1b). The distance between two slide glasses is the same as the thickness of a slide. This distance becomes actually the thickness of the solid PDMS slab.

(a) (b)

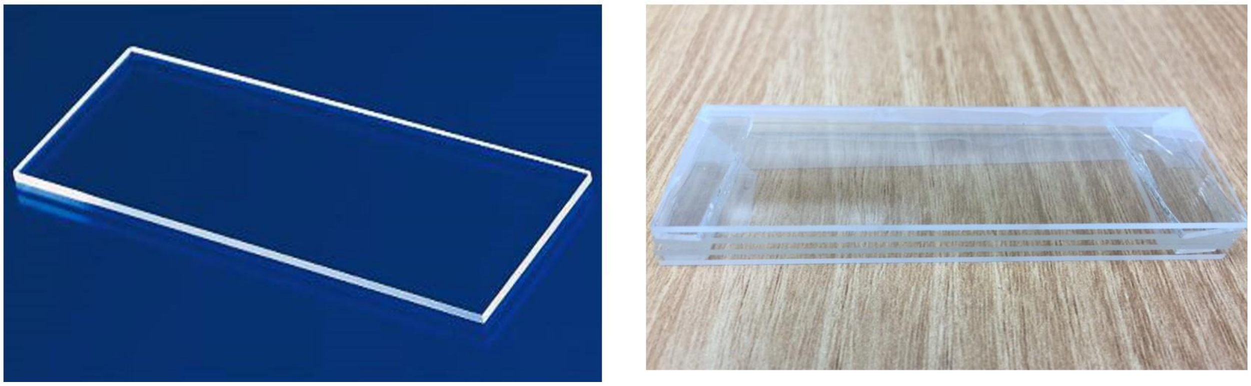

Fig. S1. (a) Fisherfinest™ Premium Plain Glass Microscope Slides. Specification of the glass Material glass; precleaned; white color; Length=75 mm; Width =25 mm; thickness=1 mm. (b) A mold for casting PDMS film made of plain glass microscope slides. The distance between two slide glasses is 1 mm.

After making the mold, mixture of prepolymers for PDMS (Curing agent and base with ratio 1:10) was poured in to the mold. Heating at 100°C is required to cure the PDMS. After 2 hours PDMS became solid, and then the mold was disassembled. The advantage of using this mold is that it is simple to make and PDMS slab has smooth surface with uniform thickness, which is very critical for precise alignment with sub-micron accuracy. The PDMS slab was punched to make a hole which was used for window, to allow the metal vapor pass through it.

b. Method to align TEM grid into the desired location

In this study the graphene FETs were supported on Si/SiO_2 substrates and bottom-gated using the SiO_2 (300 nm thermal oxide) as the gate dielectric and the p^+ doped Si substrate ($\rho \sim 3 \times 10^{-3}\ \Omega$ cm) as the gate electrode. Highly oriented pyrolytic graphite (Natural Kish graphite (Grade 300), Graphene supermarket) was used to produce graphene flakes by mechanical exfoliation of using adhesive tape (Scotch Tape, 3M, Inc). The specifications of the TEM grids are listed in the Table 1. To align TEM grid into the desired location, the TEM grid was attached at a hole (1 mm diameter) punched in a PDMS slab (1 mm thick) as shown in Fig. S3, as the PDMS is sticky to clean metallic surfaces.

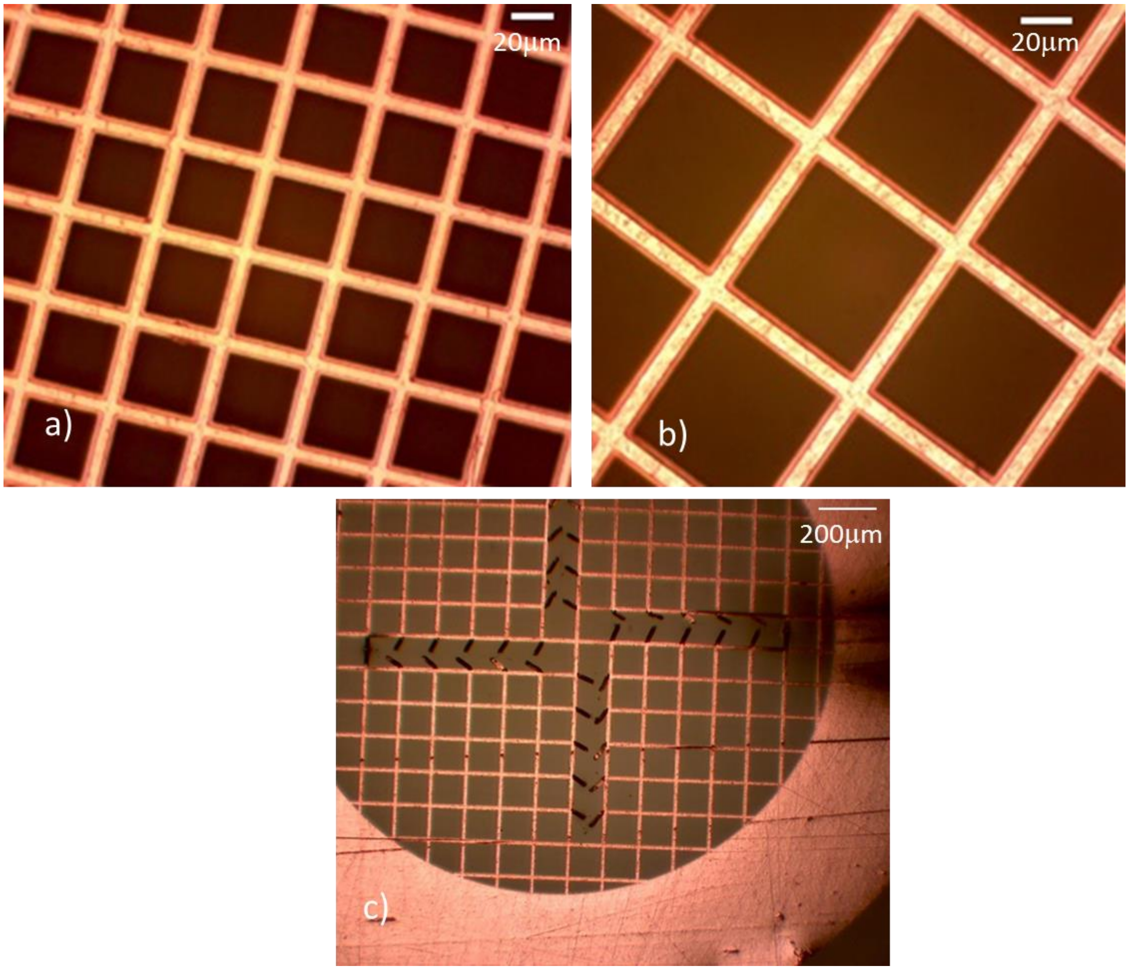

Fig. S2. (a) G600HS Fine Square Mesh TEM grid, (b) G300 Square Mesh TEM grid (magnification 600X), and (c) G300 TEM grid after cutting. The G300 TEM grid was cut to enlarge the contact pads after metal deposited.

Table 1: Specifications of the TEM grids

TEM grid	Bar width (µm)	Hole width (µm)	Remark
G600HS Fine Square Mesh	5	37	1st evaporation
G300 Square Mesh	25	58	2nd evaporation

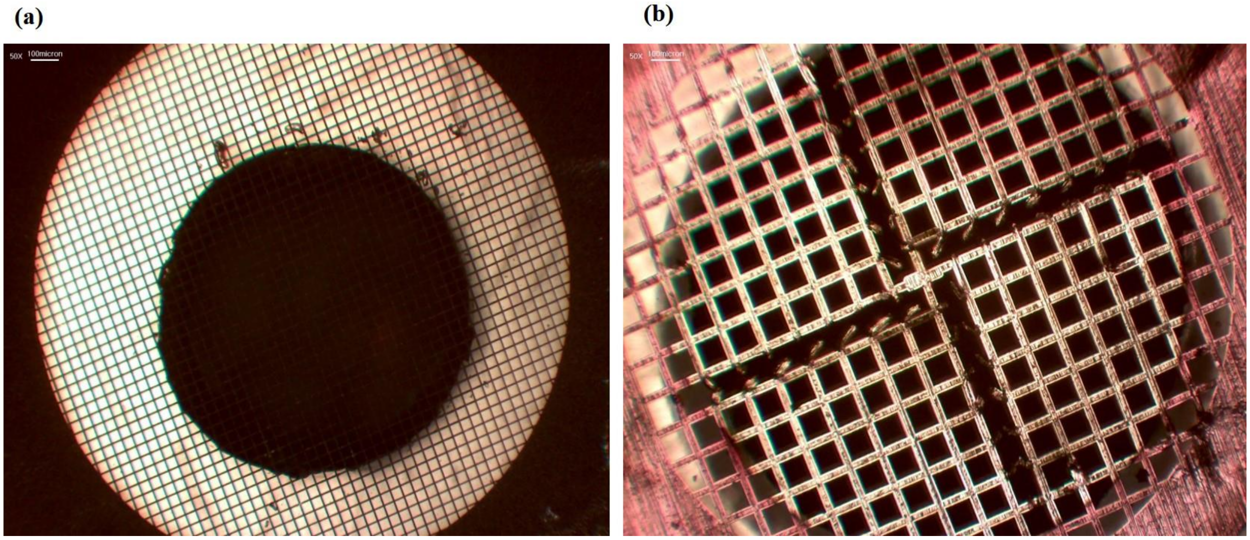

Fig. S3. (a) G600HS square mesh covering the hole of the PDMS slab. (b) G300 HS square mesh covering the hole of the PDMS slab

By using a XYZ-translator while viewing the area through an optical microscope, the TEM grid was carefully positioned in XY-plane ~3 μm above the surface of the Si wafer. After the XY-alignment, Z-directional contact was done, and the PDMS slab with grid attached to the wafer due to the adhesive property of PDMS. The adhesion was kept in high vacuum evaporation process. Ti and Au contact pads of the FET devices were evaporated onto the graphene through square holes of the TEM grid used as a shadow mask, as shown in Fig. S2.

c. PMMA residue on the device fabricated by conventional e-beam photolithography

After samples were fabricated with a conventional e-beam lithography technique, the sample was cleaned with dissolving and annealing, but the PMMA residue was not removed completely. Specifically, dissolving was performed with boiled acetone and chlorofom cleaning, and annealing was done with Ar/H_2 flow, at 400 ^oC, overnight. From AFM measurement, residue layer with 1 nm roughness was found, as shown in Fig. S4.

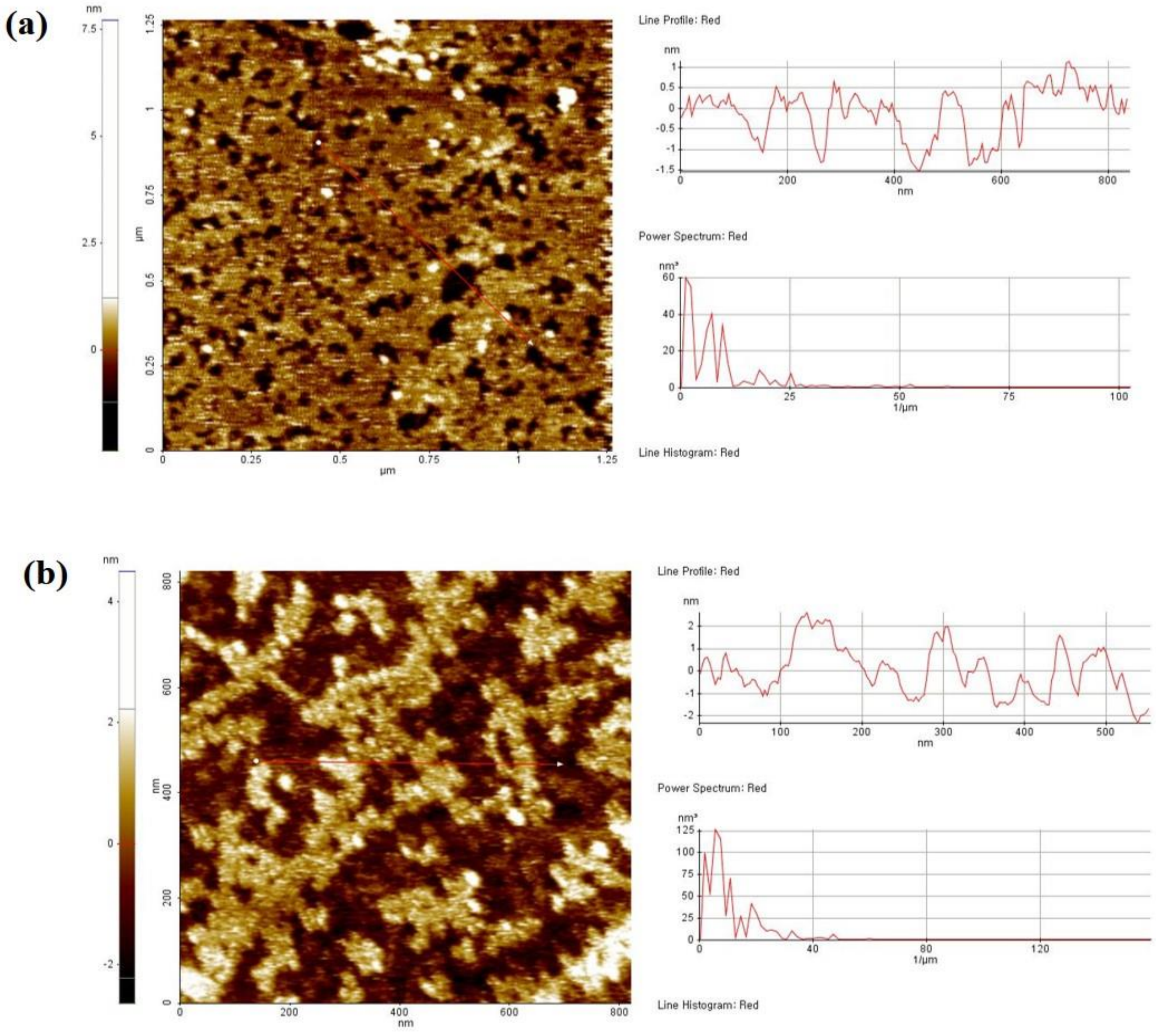

Fig. S4. (a) AFM image was taken after the sample was cleaned with boiled acetone and chloroform where black and brown areas were suspected as graphene and PMMA residue, respectively. (b) AFM image was taken after the sample was annealed in Ar/H_2 flow at 400 ^oC.

2. The resistance change depending on gate voltage for different graphene samples in different environments.

a. Graphene on hBN devices

We prepared 6 FET samples with graphene on hBN using TEM grid as a shadow mask. All of them showed the same trends in air, low vacuum, and high vacuum, as shown Fig. S5.

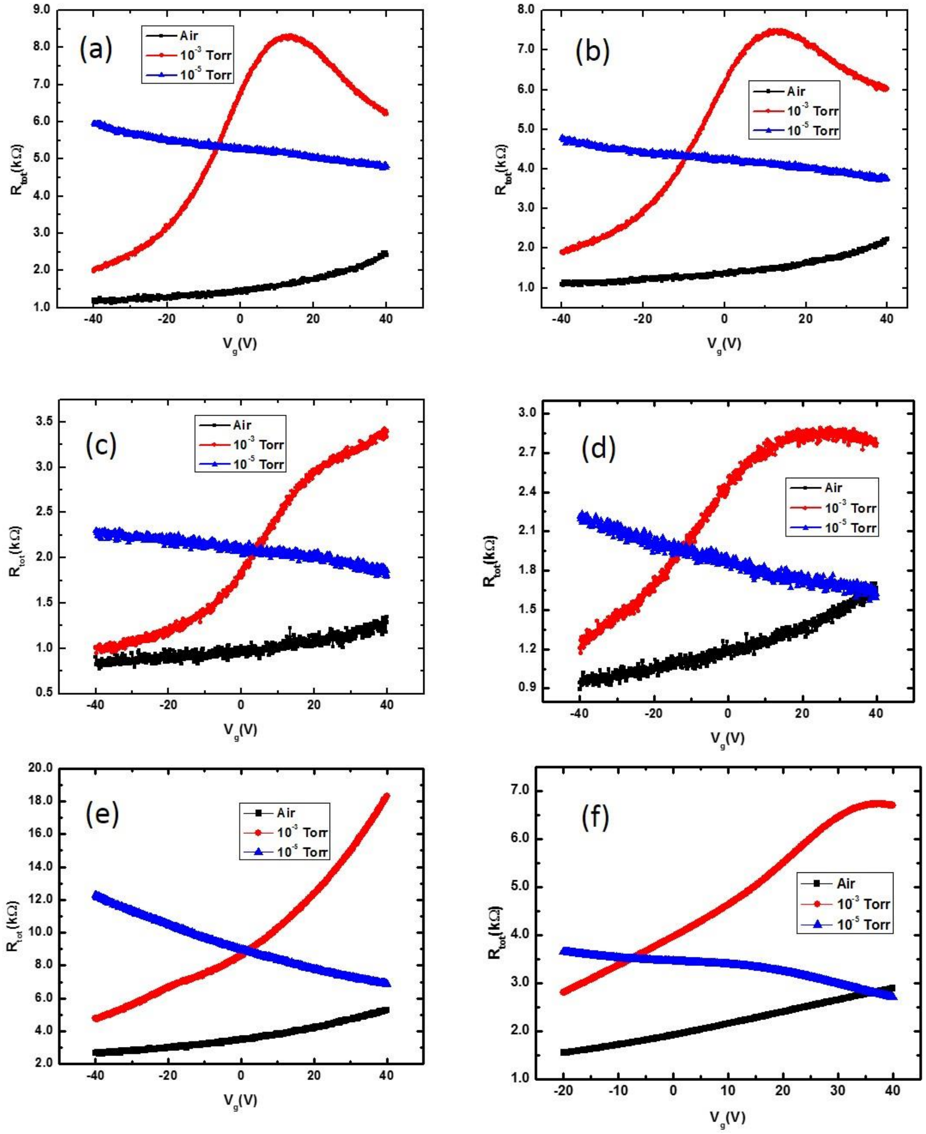

Fig.S5. The relationship between total resistance R_{tot} and back-gate bias V_g of six graphene-on-hBN devices: (a) sample#1, (b) #2, (c) #3, (d) #4, (e) #5, and (f) #6 in air and in vacuum fabricated by using TEM grids.

b. Graphene on Si devices fabricated using TEM grids

We prepared 2 FET samples with graphene on SiO_2 using TEM grid as a shadow mask. Both of them showed the same trends in air, low vacuum, and high vacuum, as shown Fig. S6.

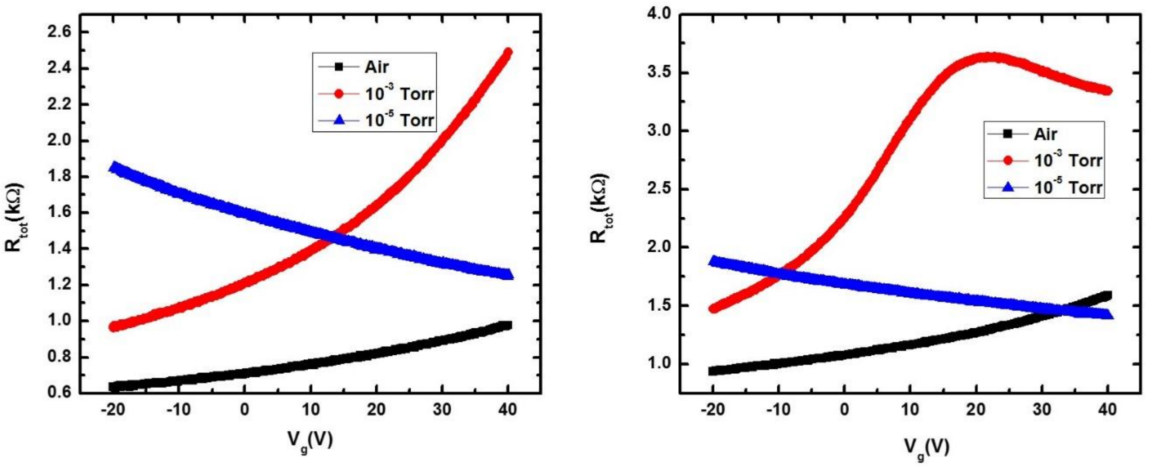

Fig. S6. The relationship between total resistance R_{tot} and back-gate bias V_g of graphene on Si devices: (a) sample#8 (b) #7 in air and in vacuum fabricated using TEM grids

c. Graphene-on-hBN devices fabricated using TEM grids in liquid environment

Graphene-on-hBN device also showed the same results as graphene-on-SiO_2, either in water or in ethanol (Fig. S7). Therefore, the behavior in liquid does not depend on the substrates.

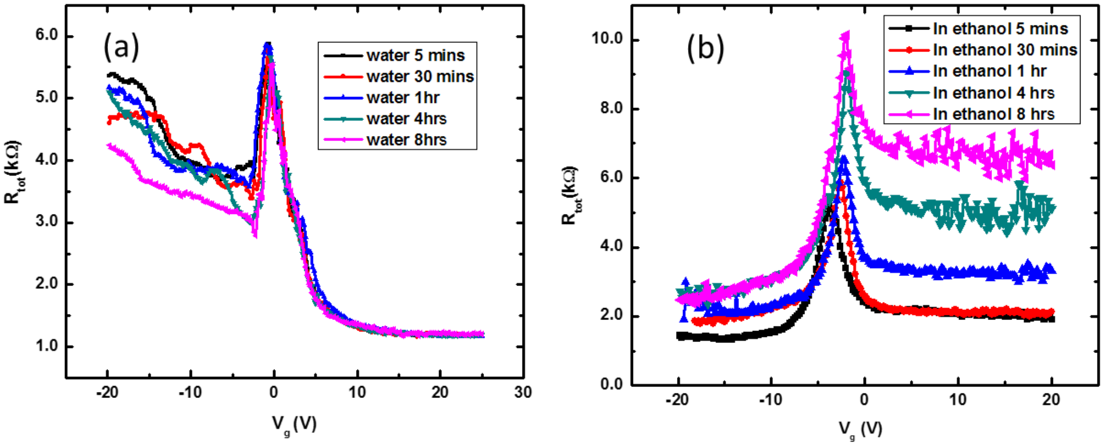

Fig. S7 The relationship between total resistance R_{tot} and back-gate bias V_g of the graphene on hBN device fabricated using TEM grids; (a) in water; (b) in ethanol. All measurements were carried out at room temperature.

d. Extrapolating and estimation of Dirac peaks

Dirac points were estimated from extrapolating the data in Fig. 2(a) into Fig. 2(d). From the data in Fig. 2(d), it is confirmed that the shape and baseline of curves are the same, and only the shifts in x-axis can be found. As the R_{tot} at V_g= 40V in air in Fig. 2(a) have lower values than the R_{tot} at V_g= -20V measured at 7hr in Fig. 2(d), the Dirac point in air in Fig. 2(a) should be shifted to about 80 V. By the same manner, the Dirac points at 10^{-3} and 10^{-5} Torr in Fig. 2(a) are estimated to be about 60, -30 V, respectively. These estimation from extrapolation is added in this revision.

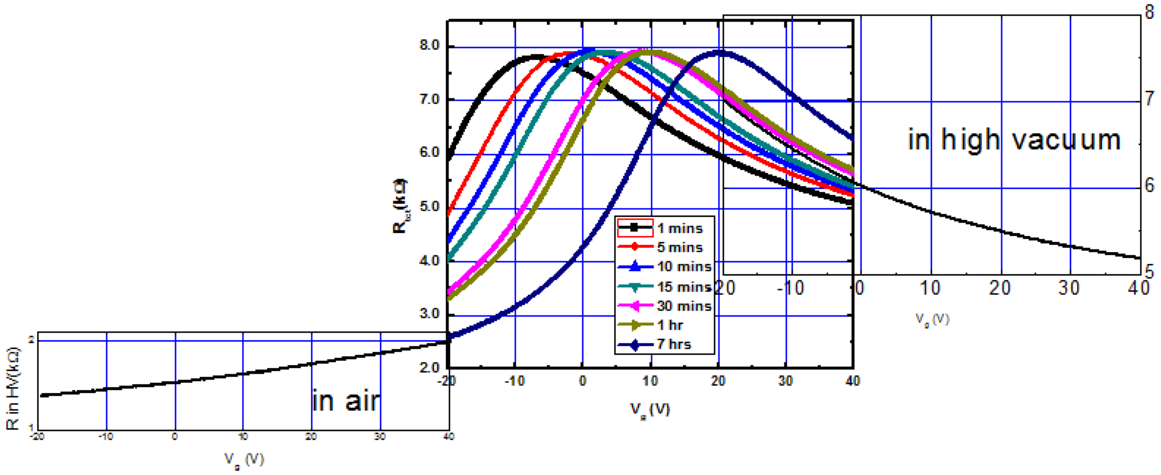

Fig. S8 While the shape and baseline of curves in the data in Fig. 2(a) and (d) were similar, but the shifts in x-axis were found. Dirac points were estimated from extrapolating the data in Fig. 2(a) into Fig. 2(d).

e. Time evolution of Dirac peak shift

The time evolution of Dirac point was plotted, as shown in Fig. S9. The tendency of our result is similar to previously published result,[1-4] but the time constant estimated from exponential function fitting was much shorter (time constant = 0.76 hr). This fast adsorption can be attributed to the clean surface of the FET using shadow mask.

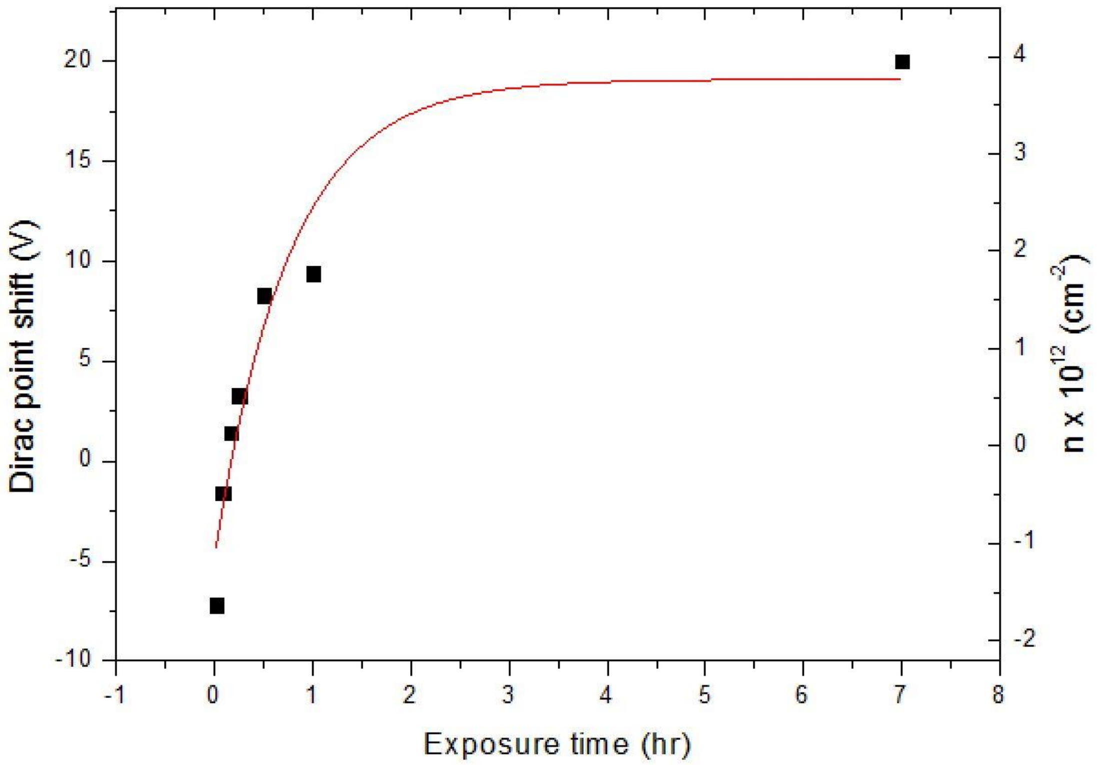

Fig. S9 Time evolution of Dirac point was fitted to an exponential function, and time constant was estimated as 0.76 hr.

f. Sticky residue on graphene

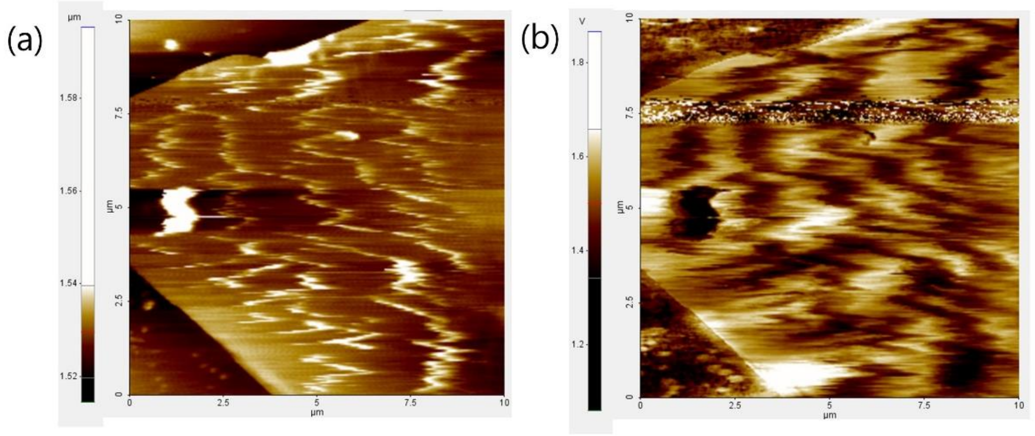

Fig. S10 In order to confirm the adhesive being washed by ethanol, graphene sample was exfoliated with the Scotch tape, and submerged into ethanol. (a) Particles and residues were found on graphene surface from AFM topography. (b) Particularly, strong frictional forces from LFM images were found on graphene surface. This means that the sticky residue on SiO_2 was dissolved by ethanol, and some amount of this residue was moved onto the graphene surface.

References

1 Levesque, P. L. *et al.* Probing Charge Transfer at Surfaces Using Graphene Transistors. *Nano Lett* **11**, 132-137, doi:10.1021/nl103015w (2011).

2 Shim, J. *et al.* Water-Gated Charge Doping of Graphene Induced by Mica Substrates. *Nano Lett* **12**, 648-654, doi:10.1021/nl2034317 (2012).

3 Sojoudi, H., Baltazar, J., Henderson, C. & Graham, S. Impact of post-growth thermal annealing and environmental exposure on the unintentional doping of CVD graphene films. *J Vac Sci Technol B* **30**, doi:Artn 041213

10.1116/1.4731472 (2012).

4 Joshi, P., Romero, H. E., Neal, A. T., Toutam, V. K. & Tadigadapa, S. A. Intrinsic doping and gate hysteresis in graphene field effect devices fabricated on SiO2 substrates. *J Phys-Condens Mat* **22**, doi:Artn 334214

10.1088/0953-8984/22/33/334214 (2010).
